# Adoption and use of social media among public health departments

**DOI:** 10.1186/1471-2458-12-242

**Published:** 2012-03-26

**Authors:** Rosemary Thackeray, Brad L Neiger, Amanda K Smith, Sarah B Van Wagenen

**Affiliations:** 1 Department of Health Science, Brigham Young University, 221 Richards Building, Provo, UT, USA

## Abstract

**Background:**

Effective communication is a critical function within any public health system. Social media has enhanced communication between individuals and organizations and has the potential to augment public health communication. However, there is a lack of reported data on social media adoption within public health settings. The purposes of this study were to assess: 1) the extent to which state public health departments (SHDs) are using social media; 2) which social media applications are used most often; and 3) how often social media is used interactively to engage audiences.

**Methods:**

This was a non-experimental, cross sectional study of SHD social media sites. Screen capture software Snag-It^® ^was used to obtain screenshots of SHD social media sites across five applications. These sites were coded for social media presence, interactivity, reach, and topic.

**Results:**

Sixty percent of SHDs reported using at least one social media application. Of these, 86.7% had a Twitter account, 56% a Facebook account, and 43% a YouTube channel. There was a statistically significant difference between average population density and use of social media (p = .01). On average, SHDs made one post per day on social media sites, and this was primarily to distribute information; there was very little interaction with audiences. SHDs have few followers or friends on their social media sites. The most common topics for posts and tweets related to staying healthy and diseases and conditions. Limitations include the absence of a standard by which social media metrics measure presence, reach, or interactivity; SHDs were only included if they had an institutionally maintained account; and the study was cross sectional.

**Conclusions:**

Social media use by public health agencies is in the early adoption stage. However, the reach of social media is limited. SHDs are using social media as a channel to distribute information rather than capitalizing on the interactivity available to create conversations and engage with the audience. If public health agencies are to effectively use social media then they must develop a strategic communication plan that incorporates best practices for expanding reach and fostering interactivity and engagement.

## Background

Social media has enhanced communication between individuals and organizations and it has the potential to augment public health communication. Social media refers to "activities, practices, and behaviors among communities of people who gather online to share information, knowledge, and opinions using conversational media" [1]. Social media applications are broadly categorized as forums and message boards, review and opinion sites, social networks, blogging and microblogging, bookmarking, and media sharing [2]. In particular, social media allows organizations to talk to their customers, for customers to talk to each other, and for customers to talk to the organization [3]. In public health, social media can be used to inform, educate, and empower people about health issues [4], to enhance the speed at which communication is sent and received during public health emergencies or outbreaks [5], to mobilize community partnerships and action [6], to facilitate behavior change [7], to collect surveillance data [8], and to understand public perceptions of issues [9].

Individual, corporate and organizational use of social media is increasing. It is estimated that by 2015, the number of individuals and corporations who have social networking accounts will reach over three billion [10]. Sixty-five percent of adult internet users in the U.S. use social networking sites [11]. Technorati currently registers over 1.2 million blogs [12]. Thirteen percent of internet users have a Twitter account [13]. A study among Fortune 500 companies reported that 60% of corporations had Twitter accounts and 56% had Facebook profiles [14]. Among Forbes 200 largest charities, use of at least one form of social media increased from 75% in 2007 to 97% in 2009 [15]. In public health, the Centers for Disease Control and Prevention is actively using social media [16]. However, there is a lack of data on social media adoption within broader public health settings, particularly state public health departments (SHDs).

A SHD baseline measure of social media adoption can serve as a benchmark for how public health agencies are doing at increasing access to health information through technology, an objective identified in Healthy People 2020 [17]. These data may act as a catalyst to accelerate social media use and encourage more research on the effectiveness of social media in public health. Therefore, the purposes of this study were threefold: 1) to assess the extent to which SHDs are using social media; 2) to determine which social media tools are used most often; and 3) to assess the extent to which social media is being used interactively to engage audiences.

## Methods

This was a non-experimental, cross sectional study. We gathered SHD website URLs from the *National Public Health Information Coalition *list [18]. We considered the SHD to be using social media if the website home page indicated an institutionally maintained account for at least one of five social media applications. SHDs were excluded if the social media application did not represent the entire SHD (e.g., Twitter account exclusively for flu response). We used screen capture software, Snag-It^®^, to obtain screenshots of each social media homepage and related content for one month (February 2011 - March 2011). This software captures a screen image and archives it as an electronic file. Two researchers independently coded each of the screenshots for three areas: presence, interactivity, and reach (Table 1, Table 2). The post and tweet topics were categorized based on the classification scheme of the Centers for Disease Control and Prevention website homepage. Inter-rater coding agreement occurred 98.7% of the time. Researchers resolved discrepancies by jointly reviewing the screenshot and re-coding the variable.

**Table 1:** Fields included on the coding sheet

Facebook	Twitter	YouTube	Flickr
Facebook (FB) page URL	Twitter URL	Youtube URL	Flickr URL

Number of people who like the FB page	Number of tweets in one month & total all-time tweets	Date YouTube channel was established/joined	Date joined Flickr

FB Photos (yes/no) & number of photos	Number of Twitter followers	Number of YouTube upload views	Number of photos in photo stream

Date of first/last FB wall post	Number of Twitter users the state is following	Number of YouTube subscribers	Number of photo sets/photos in each set

FB discussions (yes/no) & number of discussions	Date of the first/last tweet	Number of YouTube channel views	Number of photo set views

FB events (yes/no) & number of events	Total re-tweets	Number of YouTube videos posted	Number of comments on each photo set

FB videos (yes/no) & number of videos	Total @ symbols (replies or responses)	Name of YouTube video	Date of photo set

Date of the individual FB wall post	Total hash-tag symbols	Date YouTube video was posted	

Internal or external post (yes/no)	Post tweet via third-party API	Number of YouTube video views	

Post an auto feed (yes/no)		Number of YouTube video likes/dislikes	

FB post original (yes/no)		Number of YouTube video comments	

Total number of comments on FB individual post			

Total number of likes on FB individual post			

**Table 2:** Metrics for coding state health department use of social media*

Metric	Definition	Twitter metric	YouTube metric	Flickr metric	Facebook metric
Presence	existence of particular social media feature	number of tweets	number of videos	number of photos	number of posts, videos, views, events, discussions, notes, & videos

Interactivity	audience members posting content, comments, or likes	tweets, replies to tweets	comments, likes, dislikes	comments	comments, likes, dislikes, posts, replies to posts, comments

Reach	number of people the SHD connected with through the social media application	number of followers	number of subscribers	number of views	number of page likes

*Applications were selected for inclusion based on data obtained from Quantcast, Compete, and Alexa, that indicated these are the most commonly used within each social media category

## Results

Sixty percent (n = 30) of SHDs were using social media. Twenty-two percent (n = 11) of SHDs used one social media application and 22% (n = 11) used two, while 8% (n = 4) were using three or four applications. Among SHDs using at least one social media application, 86.7% (n = 26) had a Twitter account, 56% had a Facebook account (n = 17), 43% (n = 13) had a YouTube channel and 13% (n = 4) had a Flickr account. Only one SHD had a blog. The reach of social media varied by each application (Table 3, Table 4, Table 5). The mean number of people who liked a SHD Facebook page was 789; SHDs had an average of 983 Twitter followers; the mean number of YouTube subscribers was 40.

**Table 3:** Facebook metrics associated with state health departments

State	Page Established	# of Page Likes	Total Posts in one Month	Posts with Likesno (%)	Total Likes on all Posts	Posts with Commentsno. (%)	Total Comments on Posts	Ratio of Posts to Comments	Engagement Rate*
AL	7/30/2009	2 227	93	67 (72)	117	8 (9)	19	11.63	.066

AK	6/1/2009	1 066	17	16 (94)	68	6 (36)	9	2.83	.072

AZ	7/2/2009	847	84	27 (32)	56	7 (8)	11	12.00	.067

AR	4/5/2010	745	9	7 (78)	28	2 (22)	6	4.50	.046

CA	6/29/2009	1 511	24	22 (92)	120	11 (46)	21	2.18	.224

CO	1/19/2010	247	19	12 (63)	17	3 (16)	6	6.33	.093

CT	6/25/2009	557	54	7 (13)	9	1 (2)	1	54.00	.018

HI	3/30/2010	132	15	2 (13)	3	0 (0)	0	0.0	.023

LA	10/1/2010	1 179	63	46 (73)	101	14 (22)	40	4.50	.111

MI	1/23/2009	1 993	23	14 (61)	41	3 (13)	6	7.67	.024

MS	10/20/2010	76	2	1 (50)	1	1 (50)	2	2.00	.039

NY	8/11/2010	432	30	21 (70)	61	4 (13)	10	7.50	.164

OH	11/16/2009	678	16	12 (75)	20	2 (13)	5	8.00	.037

RI	10/27/2010	81	27	15 (56)	50	4 (15)	9	6.75	.728

TN	12/11/2009	1 289	9	3 (33)	7	1 (11)	2	9.00	.007

VT	12/2/2010	120	6	2 (33)	3	0 (0)	0	0.0	.025

WA	7/22/2010	231	15	3 (20)	5	2 (13)	4	7.50	.039

* Engagement rate = *likes *+ *comments*/number of page fans

**Table 4:** Twitter metrics associated with state health departments

State	Twitter	Twitter followers	All-time total tweets	Tweets in one month	@ Replies one month	Re-tweets in one month
AK	1/30/2009	1 404	778	43	0	22

AZ	3/24/2009	2 284	976	104	0	1

AR	5/9/2010	181	36	4	0	0

CA	4/21/2009	3 039	802	43	0	2

CT	4/27/2009	1 394	396	47	2	20

DE	6/15/2009	714	76	1	0	0

HI	10/8/2009	1154	1 572	159	7	120

IN	8/9/2009	79	21	1	0	0

IA	4/30/2009	2 609	292	48	0	3

KS	9/2/2009	324	145	2	0	1

LA	9/3/2010	759	553	63	3	13

MA	4/12/2010	1 502	296	17	0	0

MI	7/16/2009	1 841	85	4	0	0

MN	3/26/2009	1 238	445	3	0	0

MS	10/3/2008	624	674	6	0	0

MO	10/19/2009	70	42	4	0	1

NH	5/21/2010	73	8	8	0	0

NJ	2/14/2011	172	151	17	1	0

NY	4/8/2010	467	253	12	0	0

OH	11/16/2009	1 385	304	11	0	6

RI	4/25/2009	218	33	14	0	0

SC	1/4/2011	473	124	4	0	0

TN	12/11/2009	552	377	11	0	0

VT	4/27/2009	114	125	18	0	3

VA	9/8/2010	1 511	577	22	0	0

WA	7/23/2009	1 367	2 853	189	0	0

**Table 5:** YouTube metrics associated with state health departments

State	Channel established	Number of subscribers	Number of videos	Uploaded views	Videos with likes no.(%)	Number of likes	Number of comments
AL	3/22/2010	7	40	12 144	2 (5)	5	10
AZ	4/21/2008	282	146	24 872	46 (32)	1 100	22
AR	5/7/2010	2	75	75	1 (1)	1	0
CA	8/22/2008	99	56	51 142	9 (16)	23	4
CO	8/16/2010	2	15	1 547	0 (0)	0	0
IN	6/17/2010	3	4	201	0 (0)	0	0
LA	11/7/2010	2	18	2 362	3 (17)	7	1
MO	10/14/2010	12	32	7 079	3 (9)	3	1
NY	2/21/2007	27	9	45 259	8 (88)	32	7
NC	7/1/2008	38	50	31 308	9 (18)	25	0
OH	4/29/2009	4	6	554	1 (17)	1	0
VT	1/14/2010	4	14	1 509	2 (14)	2	1

Posting on social media sites averaged once per day (Table 3, Table 4). Twitter re-tweets constituted 22.5% of all tweets; only 1.5% of tweets were in response to a tweet made by a follower. The SHD was the primary author of nearly all Facebook posts (89.5%). Just over a quarter (26.9%) of Facebook posts were an auto-feed, meaning that the content was originally posted on a third-party API (e.g., HootSweet). The majority (86%) of Facebook posts received no comments and 45.1% of Facebook posts had no likes (Table 3).

The majority of Twitter tweets (79.7%) were health related, 14.1% were non-health related, and for 6.2%, the information was not adequate to determine the topic area. The health-related tweets focused on general areas of staying healthy (39.7%), diseases and conditions (26.2%), environmental health (8%), injury, violence, and safety (5.4%), emergency preparedness and response (4.6%), other diverse areas (16.1%). Specifically, the most common topics were nutrition (11.4%), heart disease (7.3%), cancer (7.3%), environmental health (6.5%), tobacco use (6.5%), flu, (5.4%) and emergency preparedness and response (3.8%).

The majority of Facebook posts (88.3%) were health-related. Non-health posts had no common threads and included topics such as deaths of prominent officials, business awards, office hours, and job openings. Of the health-related posts, 77.8% were factual health-related information, 6.8% were about services offered, and 15.7% were event announcements. The most common topics were flu (9.5%), environmental health (6.0%), heart disease (5.5%), nutrition (4.5%), tobacco (4.3%), emergency preparation (4.3%), and cancer (3.6%).

Views of photos and videos were limited. The total number of photos posted on Flickr was 167 (mean = 41.75). The ratio of views to photos ranged from 1.55 to 47.65. The mean number of You Tube videos was 30. The mean number of video views was 687 (median = 144), not including an outlier CPR-related video receiving four million views. Over three-fourths of videos (78.3%) received no likes and 70.7% of videos received no comments (Table 5).

Social media use differed between rural states and urban states. There was a statistically significant association between a SHD use of any social media and the U.S. Census Bureau's measure of the state's average population density per square mile (*r *= .346; p = .01). There were no significant differences between U.S. census region and use of social media (chi square = 2.279, p = .517).

## Discussion

The purpose of this study was to assess the degree to which SHDs were using social media and how they used it. The majority of SHDs are using at least one social media application with rates similar to large companies [14], charities [15], and nonprofit organizations [19]. However, compared to these organizations, a greater percentage of SHDs used Twitter. The overwhelming preference for Twitter may be associated with keeping the public up-to-date with SHD-related news. Yet Twitter is used by less than 13% of internet users [13], indicating a mismatch with audience preference for receiving information.

SHD's social media use varied by population density. These findings are in contrast to previous research that found no difference in individual use of social networking sites by urban or rural location [11]. The results are similar to a study that showed rural hospitals used social media less frequently than urban hospitals [20]. Additionally, on a typical day, people living in rural areas are less likely than urban residents to visit a video sharing site [21] and only 9% of Twitter users live in rural areas [22].

Audience reach with social media was limited. Relative to a state's population, the proportion of people who comprised followers, friends, and subscribers was small. An audience member's demographic characteristics, including occupation or professional affiliations, are unknown on social media applications. It is possible that the audience is the general population, or other public health professionals, including SHD employees.

Social media is more than another communication channel. As mentioned previously, there are several ways SHDs can use social media. If utilized effectively, social media has the potential to improve the way public health agencies engage, interact and communicate with its various audiences. Specifically, social media are technologies that facilitate opportunities for engaging with the audience [1] and for creating and maintaining relationships [23]. If public health agencies can use social media to engage their audiences and create relationships, something that has previously been hindered by time and distance restrictions, then they are one step closer to establishing true community-based partnerships to address public health problems.

This study showed that, SHDs are not capitalizing on social media's interactive potential. Their one-way social media communication pattern is similar to the results of an analysis of politicians and government agency Twitter posts that revealed the most common purpose was a one-way sharing of public information [24]. Very few of the audience members were viewing the videos or photos. Using comments and likes as a proxy measure for reading posts, relatively few engaged in reading. A like indicates that a person has at least read a post or watched a video, and while there was a greater proportion of likes than comments received, it is only part of the engagement process. Research shows that if a person likes a product page, they are more likely to buy the brand, recommend the brand to others and share branded content [25]. However, liking the page does not result in purchasing the product. This may be true for public health as well. Liking a page or a post may not equal following behavioral recommendations or participation in public health programs.

There may be a few reasons why SHDs have limited social media interaction. The first may be that there is a mismatch between the content that is posted and audience preferences. SHDs are posting and tweeting about health topics and not about the agency. The health topics may be a reflection of the national health observances that were occurring during February and March, including American Heart Month, National Nutrition month, National School Breakfast Week, and Colorectal Cancer Awareness Month. If the audience is primarily other health professionals, general health content may generate fewer comments. If the audience is the general public, the content may be poorly developed or the topics may be of little interest. The majority of Facebook posts were auto feeds, meaning there was little thought given to matching the content with audience preferences for information. SHDs cannot assume that because they post content on a social media application that people will respond. It is important to communicate information in a way that reflects the audience preferences, stimulates response or discussion, and is tailored to the social media application.

Public health agencies use of social media is in the early adoption stages. Because social media use is becoming so pervasive, it seems prudent for SHDs to strategically consider how to use it to their advantage. To maximize social media's potential, public health agencies should develop a plan for incorporating it within their overall communication strategy. We recommend a framework posted by Bernoff and Li as a starting point [23]. The agency must identify what audience they are trying to reach, how that audience uses social media, what goals and objectives are most appropriate, and which social media applications fit best with the identified goals and objectives.

Some study limitations should be noted. First, there is not a universally accepted standard for which social media metrics measure presence, reach, or interactivity. Second, we identified the SHD as using social media if there was an institutional account as identified on the health department website home page. It is possible that individual programs or organizational units within a health department are using social media independent of a department-wide coordinated effort. Lastly, this was a cross-sectional study to establish a baseline of social media use by SHDs.

## Conclusions

Most SHDs have recently begun to use at least one social media application. The most popular social media is Twitter despite the fact that only 13% of internet users have a Twitter account. The reach of social media is limited as evidenced by the low number of followers, page likes, and subscribers. Additionally, SHDs appear to be using social media as another channel to distribute information rather than creating conversations and engaging with the audience. If public health agencies are to use social media effectively they must develop a strategic communication plan that incorporates best practices for expanding reach and fostering interactivity and engagement.

## Competing interests

The authors declare that they have no competing interests.

## Authors' contributions

RT conceived of the study, participated its design and coordination, performed the statistical analysis, and drafted the manuscript. BLN conceived of the study, participated in its design and coordination, and helped draft the manuscript. AKS and SBV participated in the study design and coordination, collected the data, and helped draft the manuscript. All authors read and approved the final manuscript.

## Pre-publication history

The pre-publication history for this paper can be accessed here:

http://www.biomedcentral.com/1471-2458/12/242/prepub
